# A 2.5D multichannel deep learning model using contrast-enhanced ultrasound for predicting malignancy in breast nodules: a two-center study

**DOI:** 10.3389/fphys.2026.1820868

**Published:** 2026-05-01

**Authors:** Jianfeng Xie, Yan He, Jiuhe Zhu, Cuiyun Liu, Jiayuan Zhan, Lang Wang, Shaoyun Zhang, Wenjian Qin, Kun Sun

**Affiliations:** 1 Department of Ultrasound, Southern University of Science and Technology Hospital, Shenzhen, China; 2 Department of Ultrasound Medicine, Shenzhen Hospital of Southern Medical University, Shenzhen, China; 3 Shenzhen Institutes of Advanced Technology, Chinese Academy of Sciences, Shenzhen, China; 4 Department of Ultrasound, Shenzhen Baoan AirSea Hospital, Shenzhen, China

**Keywords:** breast nodules, computer-aided diagnosis, contrast-enhanced ultrasonography, deep learning, malignancy prediction

## Abstract

**Objective:**

To evaluate a novel multichannel deep learning (DL) model using contrast-enhanced ultrasound (CEUS) data with multiple regions of interest (ROIs) and time-intensity curve (TIC)-derived key frames for predicting breast nodule malignancy. Clinical features were integrated into a combined model for robust, generalizable breast lesion classification. The model was further evaluated as an AI-assisted decision support tool through direct comparison with BI-RADS classification by senior radiologists.

**Methods:**

This retrospective two-center study enrolled 141 patients with breast nodules: 89 from Institution 1 (June 2016–October 2017; training cohort, n=62; internal validation, n=27) and 52 from Institution 2 (November 2022–November 2024; external validation). BI-RADS categories were extracted from original radiology reports and binarized at ≥4B for malignancy prediction. Tumors were segmented on B-mode and CEUS images to define intratumoral ROIs, tumor bounding boxes, and peritumoral expansions (2 mm and 5 mm). TIC phases (initial, ascending, peak, descending, wash-out) were stacked into multichannel 2.5-dimensional (2.5D) inputs. DenseNet201 models, pretrained on ImageNet, were trained for 2D and 2.5D DL across ROI types. Outputs from the clinical model and optimal intratumoral plus 2-mm peritumoral ROI models were fused via logistic regression. Performance was evaluated using area under the receiver operating characteristic curve (AUC), Hosmer–Lemeshow calibration, decision curve analysis (DCA).and DeLong test for comparison with BI-RADS.

**Results:**

Among 2.5D models, the multichannel variant with intratumoral plus 2-mm peritumoral ROI showed highest external validation performance. The combined model, constructed by fusing the output of the optimal MultiChannel_2.5D_DL architecture (intratumoral + 2-mm peritumoral ROI) with the 2D_DL and clinical models via logistic regression, outperformed individual models externally (AUC 0.949 [95% CI: 0.888, 1.000] vs. clinical AUC 0.821 [95% CI: 0.671, 0.970], p=0.04; vs. 2D AUC 0.789 [95% CI: 0.660, 0.918], p=0.01; vs. 2.5D AUC 0.824 [95% CI: 0.677, 0.972], p=0.03). In direct comparison in the external validation cohort, the combined model demonstrated diagnostic performance comparable to that of senior radiologists (AUC 0.949 [95% CI: 0.888, 1.000] vs. 0.897 [95% CI: 0.808, 0.986], p=0.15).

**Conclusion:**

This combined model, integrating the optimal MultiChannel_2.5D_DL output with 2D_DL and clinical features, offers promising accuracy and generalizability as a decision support tool for CEUS-based breast nodule malignancy prediction, potentially assisting radiologists in reducing interobserver variability and unnecessary biopsies.

## 1 Introduction

Breast cancer remains a leading cause of morbidity and mortality among women worldwide, underscoring the need for accurate and non-invasive diagnostic tools to differentiate benign from malignant breast nodules (Giaquinto et al., 2022). Ultrasonography, particularly contrast-enhanced ultrasound (CEUS), has emerged as a valuable imaging modality due to its ability to provide real-time visualization of vascular perfusion patterns, which are often distinct between benign and malignant lesions (Wang et al., 2022). However, the diagnostic accuracy of CEUS relies heavily on subjective interpretation, which is limited by interobserver variability and the complexity of lesion characteristics (Chen et al., 2019; Zhou et al., 2020). To address these challenges, advanced computational approaches, such as deep learning, have been increasingly integrated into medical imaging to enhance diagnostic precision (Aggarwal et al., 2021; Mridha et al., 2021). Recent advances in deep learning have shown promising results in medical image analysis, particularly for the classification of breast lesions (Al-antari et al., 2020; Mahmood et al., 2022). Deep learning models, such as convolutional neural networks (CNNs), can automatically extract and learn complex features from imaging data, offering potential improvements over traditional radiologist-based assessments (Jiang et al., 2021). However, conventional two-dimensional (2D) deep learning models may not fully capture the dynamic temporal and spatial information provided by CEUS. Multichannel deep learning approaches, which incorporate multiple image frames or regions of interest (ROIs) derived from CEUS, can better leverage this dynamic information, thereby improving diagnostic performance. Nevertheless, few studies have explored the use of a DL model for the differentiation of benign and malignant breast nodules using CEUS.

This study aims to evaluate the diagnostic performance of a novel multichannel deep learning model based on CEUS data, utilizing multiple ROI types and time-intensity curve (TIC)-derived key frames, for predicting the malignancy of breast nodules. By integrating clinical features into a clinical-deep learning combined model, we seek to develop a robust and generalizable tool for breast lesion classification. The core innovation of this work lies in the proposed MultiChannel_2.5D_DL model, which employs a unique multichannel input strategy based on TIC key frames and systematically explores different ROI configurations (intratumoral and peritumoral) to optimize diagnostic performance.

## 2 Materials and methods

### 2.1 Patients

This retrospective study analyzed data from patients who underwent breast ultrasonography at Shenzhen Hospital of Southern Medical University and Southern University of Science and Technology Hospital. The Institutional Review Boards of both participating institutions approved the study, and informed consent was waived. The inclusion criteria were as follows: (a) All patients had no US contrast agent contraindication; (b) B-mode and US contrast ultrasound examinations of histopathologically confirmed breast tumors (diagnosed by core biopsy) and of benign breast tumors confirmed either histopathologically or by 2-year follow-up; (c) available clinical data. The exclusion criteria were meticulously defined as follows: (a) pregnant or lactating women; (b) any preoperative interventions or therapies (e.g., radiotherapy, chemotherapy, radiofrequency ablation) before US examination; (c) incomplete clinical or imaging data; (d) the target tumor was unclear or had no visible ROI on US images due to artifacts.

**Figure 1:**
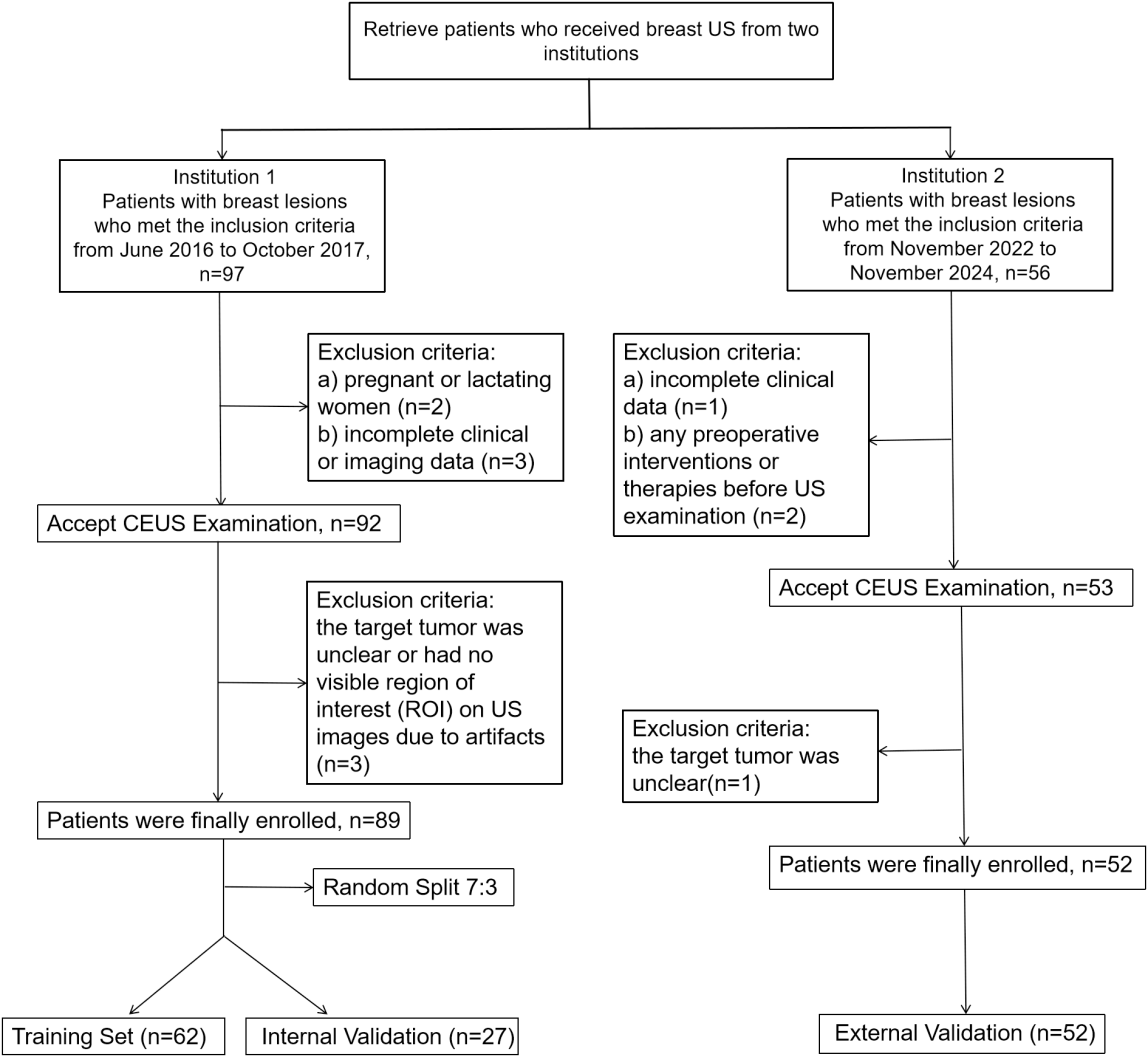
Flowchart of study inclusion and exclusion. Institution 1, Shenzhen Hospital of Southern Medical University; Institution 2, Southern University of Science and Technology Hospital; CEUS, contrast-enhanced ultrasound.

The training and internal validation datasets were obtained from Shenzhen Hospital of Southern Medical University between June 2016 and October 2017 using a Philips EPIQ5 ultrasound system, comprising a total of 89 patients with breast nodules, and were randomly split into a training cohort (n = 62) and an internal validation cohort (n = 27) at a 7:3 ratio. The external validation dataset was collected from Southern University of Science and Technology Hospital between November 2022 and November 2024 using a Mindray Nuewa R9T ultrasound system, comprising 52 patients. **Figure 1** illustrates a flowchart delineating the patient inclusion process.

### 2.2 Clinical variables

The clinical variables included in this study were patients age, maximum diameter of the breast nodule, composition, echo pattern, posterior acoustic features, aspect ratio, Boundary of enhancement, margin characteristics, calcifications, pathological data, and color Doppler flow imaging (CDFI) blood flow was graded using the Adler classification (Adler et al., 1990).

### 2.3 Imaging protocols

All examinations were conducted by sonographers with more than five years of dedicated experience in breast ultrasound, each having undergone specialized training to harmonize the interpretation of key imaging features and to ensure proficiency in using structured reporting templates. B-mode US and CEUS were obtained and archived following established protocols. Qualitative feature evaluations (e.g., assessment of margins or signs of invasion) were independently carried out by two radiologists from the breast subspecialty section of the ultrasound department, with both blinded to patient outcomes to maintain assessment consistency. Disagreements were settled through joint review with a senior radiologist. This standardized workflow guaranteed consistent recording of conventional ultrasound characteristics across the entire patient cohort. After B-mode imaging, CEUS was performed with a low mechanical index (approximately 0.06) to assess tumor perfusion. An intravenous bolus of 4.8mL sulfur hexafluoride microbubble contrast agent (SonoVue^®^) was administered, immediately followed by a 5 mL saline flush. The dynamic enhancement process of the lesion was continuously monitored and stored for 1–2 minutes. For subsequent analysis, the imaging plane displaying either the largest cross-section of the tumor or the most abundant vascularization was selected.

### 2.4 Image processing and standardization

In our study, the ROI was manually delineated on the dataset using ITK-SNAP 4.0.2, with annotations conducted by two experienced radiologists. Any discrepancies in their annotations were resolved by a senior radiologist boasting over 20 years of experience. For each patient, we selected the slice that presented the largest ROI as the representative image. Manual segmentation of tumors is performed on B-mode ultrasound images and CEUS images, we generate intratumor, tumor bounding boxes. This tumor segments was expanded by an additional 2mm, 5mm.

At Institution 1 (2016–2017), imaging was performed using a Philips EPIQ5 system with a 9 MHz linear probe, while at Institution 2 (2022–2024), a Mindray Nuewa R9T system with a 12 MHz linear probe was used. All inputs were resized to 1024 × 768 pixels and normalized using the mean and standard deviation values from the ImageNet dataset. Importantly, the multichannel 2.5D deep learning model primarily relied on relative changes in time-intensity curves across multiple intratumoral and peritumoral ROIs rather than absolute grayscale intensity values. This design inherently improves the model’s robustness to inter-manufacturer and temporal variations in ultrasound equipment.

### 2.5 Multi-channel image fusion

The ROI delineation is important for processing contrast-enhanced ultrasound data. The key issue in identifying the ROI in the raw TIC dataset is to pinpoint the critical phases of contrast dynamics. TIC patterns demonstrate that malignant lesions typically exhibit rapid wash-in followed by rapid wash-out or rapid wash-in followed by slow wash-out (Jung et al., 2021). In each patient, the contrast-enhanced ultrasound (CEUS) video was recorded continuously for 1–2 minutes and typically contained approximately 100–200 frames, depending on the frame rate and recording duration. To capture the essential dynamic enhancement patterns while reducing redundancy and computational burden, five representative key frames were manually selected by experienced radiologists according to the phases of the TIC: the initial stage, ascending stage, peak stage, descending stage, and wash-out stage. The selected key frames from these phases resulted in a collection of 2D images (five frames per patient) that were centered on the primary temporal ROI dynamics and adequately represented the perfusion characteristics critical for malignancy prediction.

**Figure 2:**
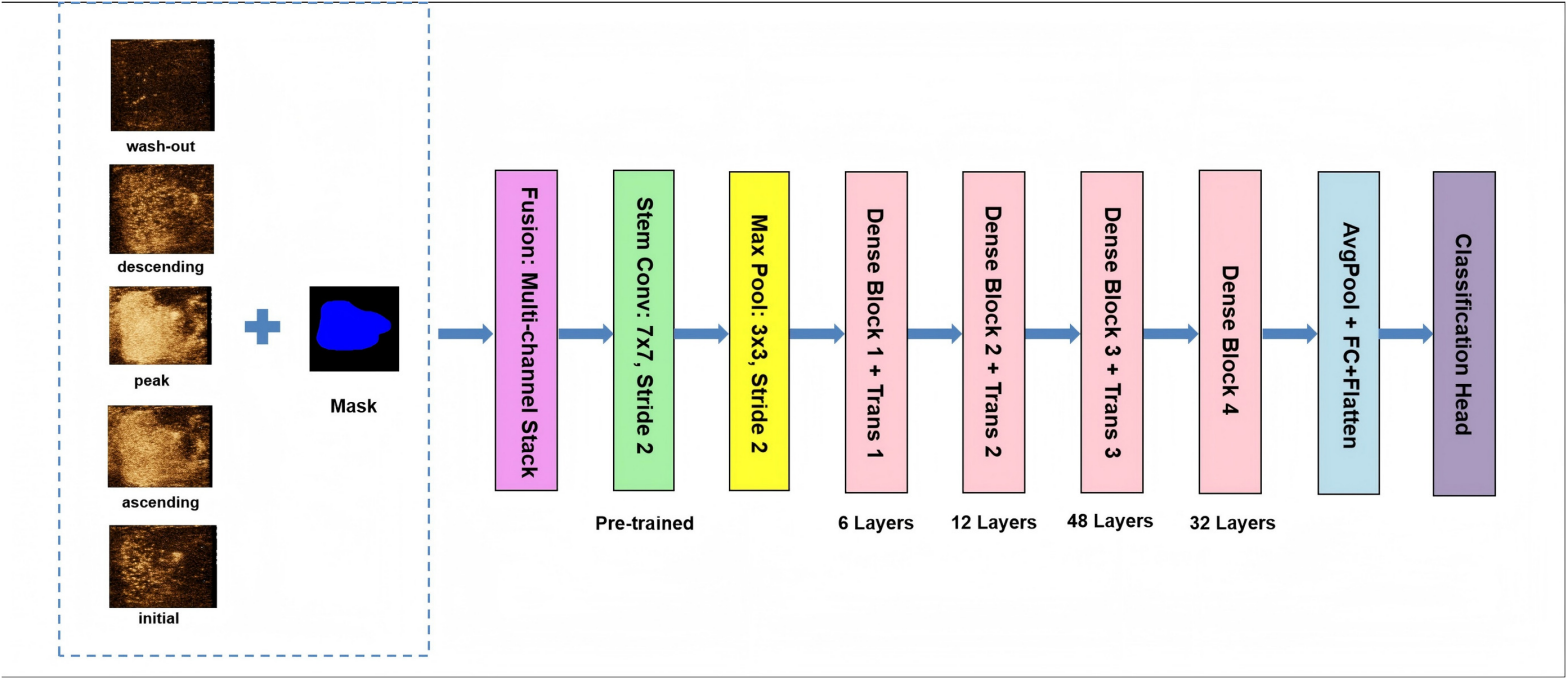
Architecture of the MultiChannel 2.5D_DL model proposed in this study. The five contrast-enhanced images on the left represent key frame slices from the contrast-enhanced ultrasound video.

Across the entire cohort of 141 patients (89 from Institution 1 and 52 from Institution 2), this process yielded a total of 705 selected key frame images (five per patient). The integration process involved stacking these frames as multi-channel 2D images to incorporate information from adjacent temporal peri-regions, which served as the final input to the deep learning model. Because the model uses multiple 2D images from various directions and, thus, could be in the middle of 2D and 3-dimensional (3D), it was called a 2.5-dimensional (2.5D) imaging technique. The model structure is shown in **Figure 2**.

### 2.6 Model training

The deep learning model was based on DenseNet201 as the backbone convolutional neural network. The model was trained end-to-end on both 2.5-dimensional inputs (multi-channel 2D images comprising five stacked key frames per patient) and standard 2D inputs. No additional data augmentation techniques were applied, and no specific measures were taken to address class imbalance.

Training was performed using stochastic gradient descent as the optimizer with cross-entropy as the loss function. The batch size was set to 32, and the model was trained for 40 epochs without early stopping. Random seeds were not fixed across experiments. All training and evaluation were conducted on a single NVIDIA GeForce RTX 4060 GPU.

For comparison, clinical models were constructed using the k-nearest neighbors (KNN) algorithm.

**Figure 3:**
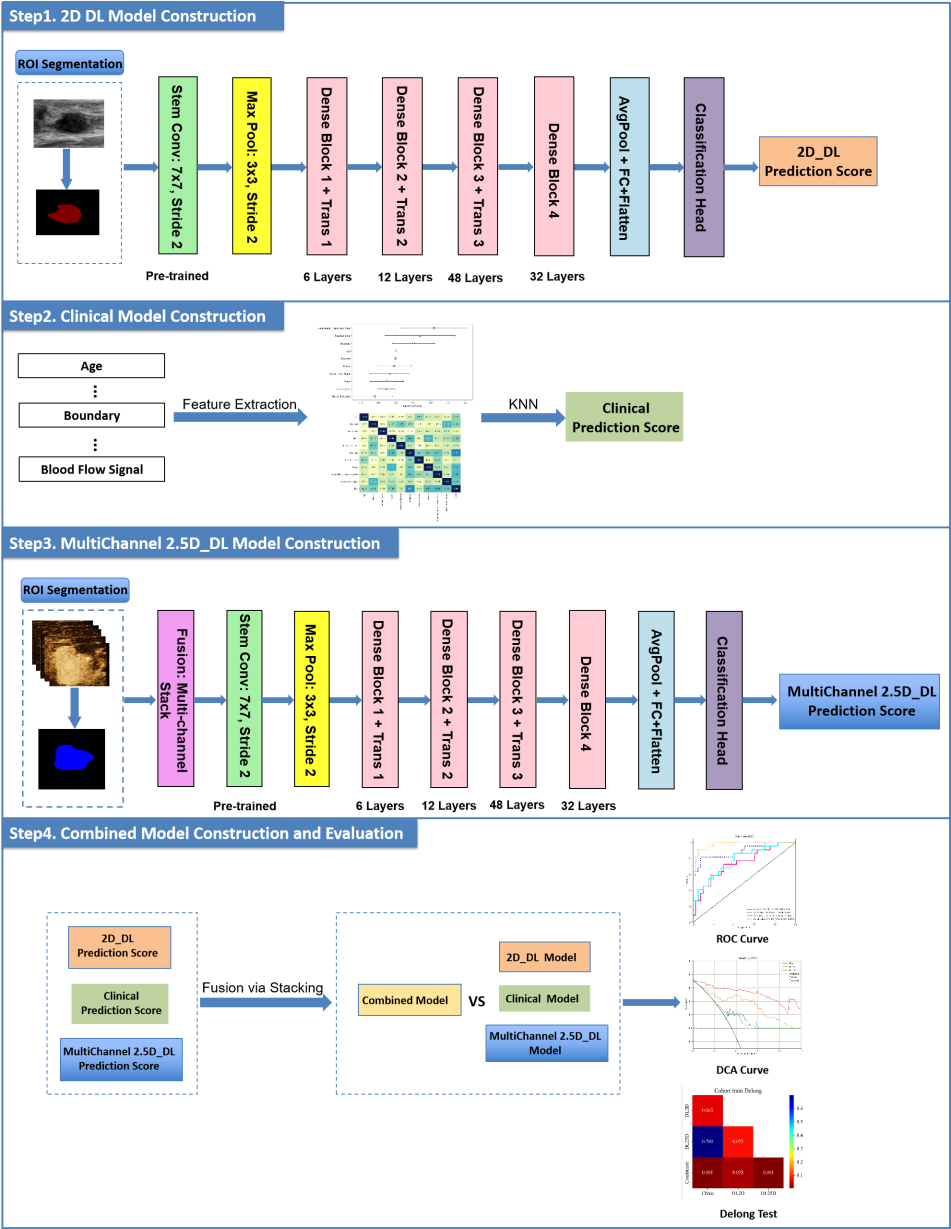
Overview of the study workflow. Steps 1–4 outline the sequential process of ROI segmentation, model training, and integration for the 2D DL, Clinical, and MultiChannel 2.5D_DL models, leading to a combined model, alongside performance evaluations via ROC curves, DCA curves, and Delong tests for assessing malignancy prediction in breast nodules. 2D 2-dimensional, 2.5D 2.5-dimensional, ROI, region of interest; DL, deep learning; ROC, receiver operating characteristic; DCA, decision curve analysis.

Finally, a combined model was developed to integrate all available predictive factors, incorporating outputs from both the 2D_DL and MultiChannel_2.5D_DL models as well as the clinical model, thereby enabling a comprehensive evaluation of enhanced predictive performance. The research flowchart is illustrated in **Figure 3**.

To visualize the image regions contributing most significantly to the model’s predictions, we employed the Gradient-weighted Class Activation Mapping (Grad-CAM) algorithm to generate heatmaps. Specifically, Grad-CAM was applied to the final convolutional layer of the model, yielding heatmaps with optimal spatial resolution.

### 2.7 Reproducibility and data availability

All key model parameters, including the DenseNet201 backbone, 2.5-dimensional multi-channel inputs, stochastic gradient descent optimizer, batch size of 32, 40 training epochs, and RTX 4060 GPU environment, are fully described in the Model Training subsection. All data analyses were executed on the OnekeyAI platform (version 4.9.1) utilizing Python 3.7.12, with statistical assessments conducted using Statsmodels version 0.13.2. Random seeds were not fixed. The complete code for preprocessing, model training, and inference is available from the corresponding author upon reasonable request.

All patient data were fully de-identified prior to analysis in accordance with the Declaration of Helsinki and institutional review board approval. No identifiable information was retained. The de-identified dataset is not publicly available due to institutional data protection policies but can be accessed from the corresponding author upon reasonable request, subject to approval by the institutional ethics committee and execution of a formal data use agreement.

### 2.8 Hyper parameters

To enhance the model’s performance across diverse patient populations characterized by substantial heterogeneity, we adopted a transfer learning paradigm. This entailed initializing the model with pre-trained weights derived from the ImageNet dataset, thereby augmenting its capacity for adaptation to varied datasets. A pivotal component of our methodology involved the judicious optimization of the learning rate to promote superior generalization across datasets. Accordingly, we utilized a cosine decay learning rate schedule, formulated as follows:


$$\eta_{t}=\eta_{min}^{i}+\frac{1}{2}(\eta_{max}^{i}-\eta_{min}^{i})(1+cos(\frac{T_{cur}}{T_{i}}\pi ))$$

where $\eta_{t\;}$is the learning rate at the current training step  t, $\eta_{min}^{i}$ and $\eta_{max}^{i}$ are the minimum and maximum learning rates in the *i*-th stage, respectively, $T_{cur}$ is the current epoch number, and $T_{i}$ is the total number of epochs in the *i*-th stage.

### 2.9 Clinical reference standard

BI-RADS categories were extracted from original radiology reports. For diagnostic performance comparison with the DL models, BI-RADS 3 and 4A were defined as benign lesions, and 4B, 4C, and 5 as malignant lesions (consistent with clinical practice for comparison with senior radiologists). All performance metrics were calculated on the external validation set.

### 2.10 Statistical analysis

Normality of continuous variables was assessed with the Shapiro–Wilk test. Group comparisons for continuous variables were performed using the independent-samples t test or the Mann–Whitney U test, as appropriate. Categorical variables were compared using the χ² test. A p-value greater than 0.05 indicated no significant difference between groups, confirming that dataset allocation was unbiased.

Model performance in the test cohort was evaluated by receiver operating characteristic (ROC) analysis, with the area under the curve (AUC) used to quantify discrimination. Calibration was assessed with calibration plots and the Hosmer–Lemeshow goodness-of-fit test. Clinical usefulness was examined using decision curve analysis (DCA) to estimate net benefit across a range of decision thresholds. Threshold probabilities ranging from 0 to 1.0 were evaluated to provide a comprehensive assessment of net benefit across the entire spectrum of possible clinical decision thresholds (Vickers et al., 2008). All data analyses were executed on the OnekeyAI platform version 4.9.1 utilizing Python 3.7.12. Statistical assessments were conducted with Statsmodels version 0.13.2.

## 3 Results

### 3.1 Patient cohort

This study enrolled 141 breast nodule patients. an age ranging from 19 to 79 years (mean age, 43.06 ± 12.16[standard deviation]). These patients were segregated into three cohorts: a training cohort (n=62), internal validation cohort (n=27), and external validation cohort (n=52). The pathological results showed 40 benign nodules and 22 malignant nodules in the training cohort, 17 benign nodules and 10 malignant nodules in the internal validation cohort, 39 benign nodules and 13 malignant nodules in the external validation cohort. Clinical data are detailed in **Table 1**. In the training cohort, significant differences were observed between benign and malignant nodules in age, shape, Boundary of enhancement, Posterior acoustic shadowing, margin and orientation (p < 0.05), which were subsequently included in clinical model. The model achieved an area under the receiver operating characteristic curve (AUC) of 0.742 (95% confidence interval (CI): 0.611, 0.874) in the training cohort. Comparable discriminative performance was observed in the external validation cohort, with an AUC of 0.821 (95% CI: 0.671, 0.970).

**Table 1:** Clinical and nodule characteristics of the study cohorts.

Clinical feature	Training cohort (62)			Internal validation cohort (27)			External validation cohort (52)		
	Benign	Malignant	P value	Benign	Malignant	P value	Benign	Malignant	P value
Age (y)	40.27 ± 11.28	48.77 ± 12.44	0.006	42.65 ± 14.37	44.00 ± 9.26	0.793	40.92 ± 11.96	48.15 ± 10.88	0.06
Lesion diameter (mm)	18.77 ± 10.25	25.00 ± 14.14	0.082	17.18 ± 6.84	19.00 ± 12.53	0.94	10.86 ± 7.84	18.23 ± 6.64	<0.001
Echo pattern			0.658			0.251			0.303
Isoechoic	2 (5.00)	1 (4.55)		1 (5.88)	0		0	0	
Mixed Echogenicity	7 (17.50)	2 (9.09)		3 (17.65)	0		1 (2.56)	2 (15.38)	
Hypoechoic	31 (77.50)	19 (86.36)		13 (76.47)	10 (100.00)		38 (97.44)	11 (84.62)	
Shape			0.028			1.0			0.025
Regular	13 (32.50)	1 (4.55)		2 (11.76)	1 (10.00)		22 (56.41)	2 (15.38)	
Irregular	27 (67.50)	21 (95.45)		15 (88.24)	9 (90.00)		17 (43.59)	11 (84.62)	
Microcalcification			1.0			0.202			0.618
Absent	9 (22.50)	5 (22.73)		9 (52.94)	2 (20.00)		26 (66.67)	7 (53.85)	
Present	31 (77.50)	17 (77.27)		8 (47.06)	8 (80.00)		13 (33.33)	6 (46.15)	
Boundary of enhancement			<0.001			0.003			<0.001
Clear	28 (70.00)	2 (9.09)		13 (76.47)	1 (10.00)		37 (94.87)	6 (46.15)	
Fuzzy	12 (30.00)	20 (90.91)		4 (23.53)	9 (90.00)		2 (5.13)	7 (53.85)	
Posterior acoustic shadowing			0.029			0.248			0.241
Absent	36 (90.00)	14 (63.64)		17 (100.00)	8 (80.00)		32 (82.05)	13 (100.00)	
Present	4 (10.00)	8 (36.36)		0	2 (20.00)		7 (17.95)	0	
Margin			<0.001			0.057			0.057
Circumscribed	17 (42.50)	0		7 (41.18)	0		12 (30.77)	0	
Non-circumscribed	23 (57.50)	22 (100.00)		10 (58.82)	10 (100.00)		27 (69.23)	13 (100.00)	
Orientation			<0.001			0.002			1.0
Parallel	36 (90.00)	10 (45.45)		16 (94.12)	3 (30.00)		34 (87.18)	11 (84.62)	
Non-parallel	4 (10.00)	12 (54.55)		1 (5.88)	7 (70.00)		5 (12.82)	2 (15.38)	
Vascularity			0.099			0.481			<0.001
Absent	21 (52.50)	6 (27.27)		7 (41.18)	2 (20.00)		26 (66.67)	1 (7.69)	
Present	19 (47.50)	16 (72.73)		10 (58.82)	8 (80.00)		13 (33.33)	12 (92.31)	

*p < 0.05, two-sided false discovery rate corrected.

### 3.2 Development and performance of deep learning models

Based on different ROI types, DenseNet201 was employed to generate 2D_DL and MultiChannel_2.5D_DL models, respectively. In the bounding box models, the MultiChannel_2.5D_DL model achieved an AUC of 0.808 (95% CI: 0.696, 0.920) in the training cohort, which was higher than that of the 2D_DL model (AUC, 0.667; 95% CI: 0.523, 0.810) (p = 0.13). however, the 2D_DL model outperformed the MultiChannel_2.5D_DL model in the internal validation cohort [AUC 0.782 (95% CI: 0.603, 0.962) vs. 0.624 (95% CI: 0.389–0.859)] (p = 0.33) and external validation cohort [AUC 0.751 (95% CI: 0.584, 0.919) vs. 0.558 (95% CI: 0.342–0.774)] (p = 0.13). In the intratumoral models, the 2D_DL model demonstrated superior performance across all validation sets compared with the MultiChannel_2.5D_DL model (mean p = 0.36). However, comparisons of different peritumoral ROI sizes in the MultiChannel_2.5D_DL models revealed that the intratumoral model combined with a 2-mm peritumoral ROI yielded the highest predictive performance. For the intratumoral model combined with the 2 mm-ROI of peritumor, the MultiChannel_2.5D_DL model (AUC, 0.776; 95% CI: 0.654, 0.898) outperformed the clinical model (AUC, 0.742; 95% CI: 0.611, 0.874) (p = 0.70) but was inferior to the 2D_DL model (AUC, 0.892; 95% CI: 0.800, 0.984) (p = 0.08) in the training cohort; in the internal validation cohort, the MultiChannel_2.5D_DL model (AUC, 0.712; 95% CI: 0.511, 0.912) outperformed both the clinical model (AUC, 0.553; 95% CI: 0.312, 0.791) (p = 0.17) and the 2D_DL model (AUC, 0.688; 95% CI: 0.466, 0.911) (p = 0.88); in the external validation cohort, the MultiChannel_2.5D_DL model (AUC, 0.824; 95% CI: 0.677, 0.972) outperformed both the clinical model (AUC = 0.821; 95% CI: 0.671, 0.970) (p = 0.96) and the 2D_DL model (AUC = 0.789; 95% CI: 0.660, 0.918) (p = 0.71). The detailed statistical results of the DL models are presented in **Table 2**.

**Table 2:** Performances of the predictive models in the study cohorts.

Model name	Set	Accuracy	AUC	Sensitivity	Specificity	PPV	NPV	F1
Intra MultiChannel_2.5D_DL	Training	0.597	0.545 (0.394-0.697)	0.727	0.525	0.457	0.778	0.561
	Internal validation	0.741	0.553 (0.295-0.811)	0.300	1.000	1.000	0.708	0.462
	External validation	0.654	0.602 (0.401-0.802)	0.538	0.692	0.368	0.818	0.437
Intra 2D_DL	Training	0.758	0.724 (0.586-0.862)	0.500	0.900	0.733	0.766	0.595
	Internal validation	0.741	0.588 (0.345-0.832)	0.500	0.882	0.714	0.750	0.588
	External validation	0.673	0.755 (0.610-0.901)	0.923	0.590	0.429	0.958	0.585
Bounding box MultiChannel_2.5D_DL	Training	0.758	0.808 (0.696-0.920)	0.864	0.700	0.613	0.903	0.717
	Internal validation	0.593	0.624 (0.389-0.859)	0.900	0.412	0.474	0.875	0.621
	External validation	0.731	0.558 (0.342-0.774)	0.538	0.795	0.467	0.838	0.500
Bounding box 2D_DL	Training	0.645	0.667 (0.524-0.810)	0.818	0.550	0.500	0.846	0.621
	Internal validation	0.741	0.782 (0.603-0.962)	0.900	0.647	0.600	0.917	0.720
	External validation	0.750	0.751 (0.584-0.919)	0.692	0.769	0.500	0.882	0.581
Intra+2mm-peri MultiChannel_2.5D_DL	Training	0.694	0.776 (0.654-0.898)	0.864	0.600	0.543	0.889	0.667
	Internal validation	0.704	0.712 (0.511 -0.912)	1.000	0.529	0.556	1.000	0.714
	External validation	0.808	0.824 (0.677 -0.972)	0.769	0.821	0.588	0.914	0.667
Intra+2 mm-peri 2D_DL	Training	0.887	0.892 (0.800 -0.984)	0.818	0.925	0.857	0.902	0.837
	Internal validation	0.741	0.688 (0.466 -0.911)	0.500	0.882	0.714	0.750	0.588
	External validation	0.692	0.789 (0.660-0.918)	0.923	0.615	0.444	0.960	0.600
Intra+5mm-peri MultiChannel_2.5D_DL	Training	0.790	0.831 (0.725-0.936)	0.727	0.825	0.696	0.846	0.711
	Internal validation	0.815	0.712 (0.478-0.946)	0.500	1.00	1.000	0.773	0.667
	External validation	0.692	0.623 (0.434-0.813)	0.538	0.744	0.412	0.829	0.467
Intra+5 mm-peri 2D_DL	Training	0.629	0.628 (0.484-0.773)	0.773	0.550	0.486	0.815	0.596
	Internal validation	0.815	0.841 (0.686-0.997)	0.900	0.765	0.692	0.929	0.783
	External validation	0.750	0.700 (0.540-0.860)	0.538	0.821	0.500	0.842	0.519
Clinical	Training	0.710	0.742 (0.611 -0.874)	0.727	0.700	0.571	0.824	0.640
	Internal validation	0.630	0.553 (0.312-0.791)	0.400	0.765	0.500	0.684	0.444
	External validation	0.808	0.821 (0.671-0.970)	0.769	0.821	0.588	0.914	0.667

2D, 2-dimensional; 2.5D, 2.5-dimensional; AUC, area under the receiver; DL, deep learning; NPV, negative predictive value; PPV, positive predictive value; F1, F1-score.

### 3.3 Combined model and performance evaluation

**Figure 4:**
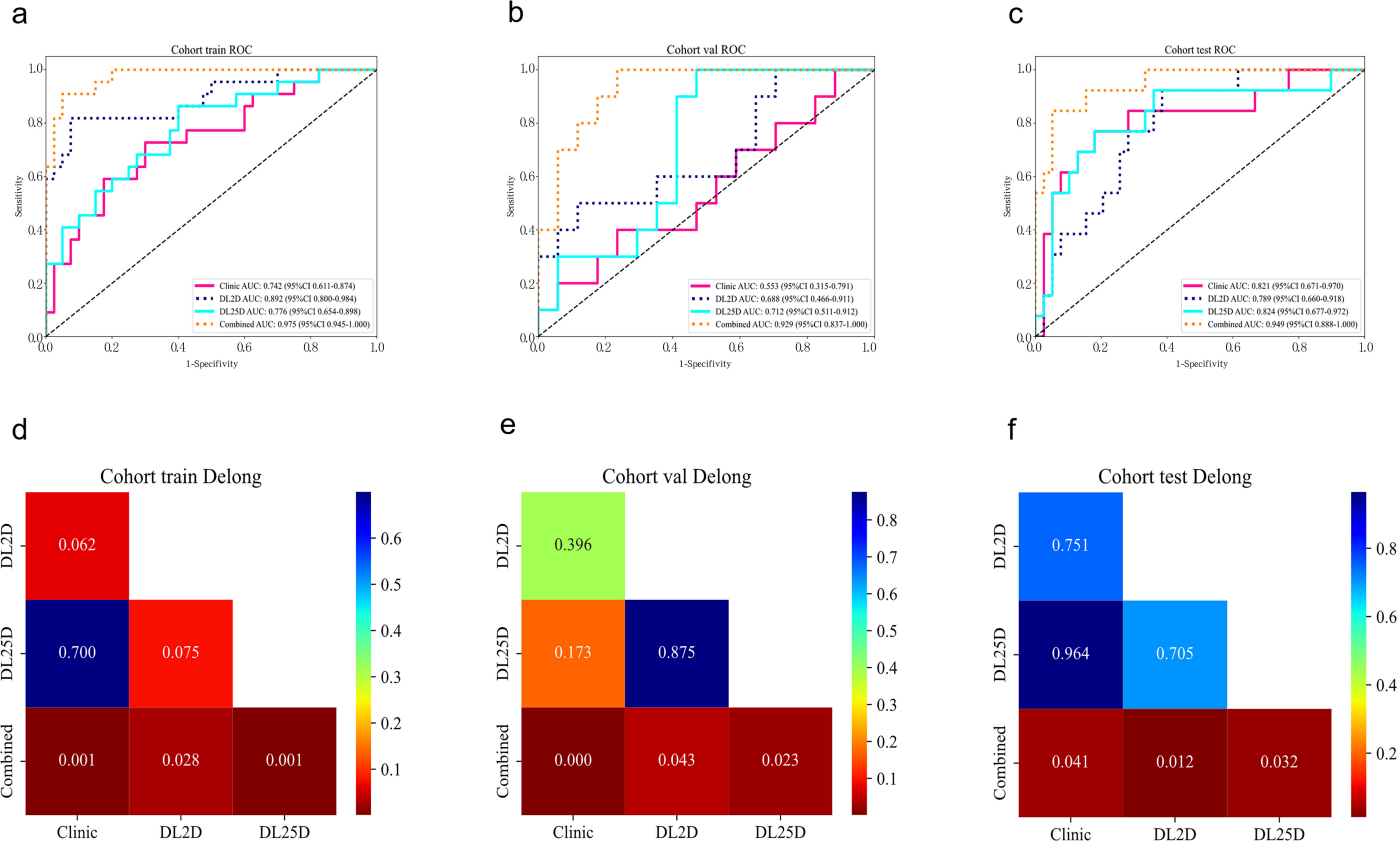
ROC curves and DeLong tests in the study cohorts. **(A–C)** illustrate the ROC curves for the models in the training, internal validation, and external validation cohorts, respectively. **(D–F)** show DeLong tests comparing the models between cohorts. 2D, 2-dimensional; 2.5D, 2.5-dimensional; ROC, receiver operating characteristic; DL, deep learning.

**Figure 5:**
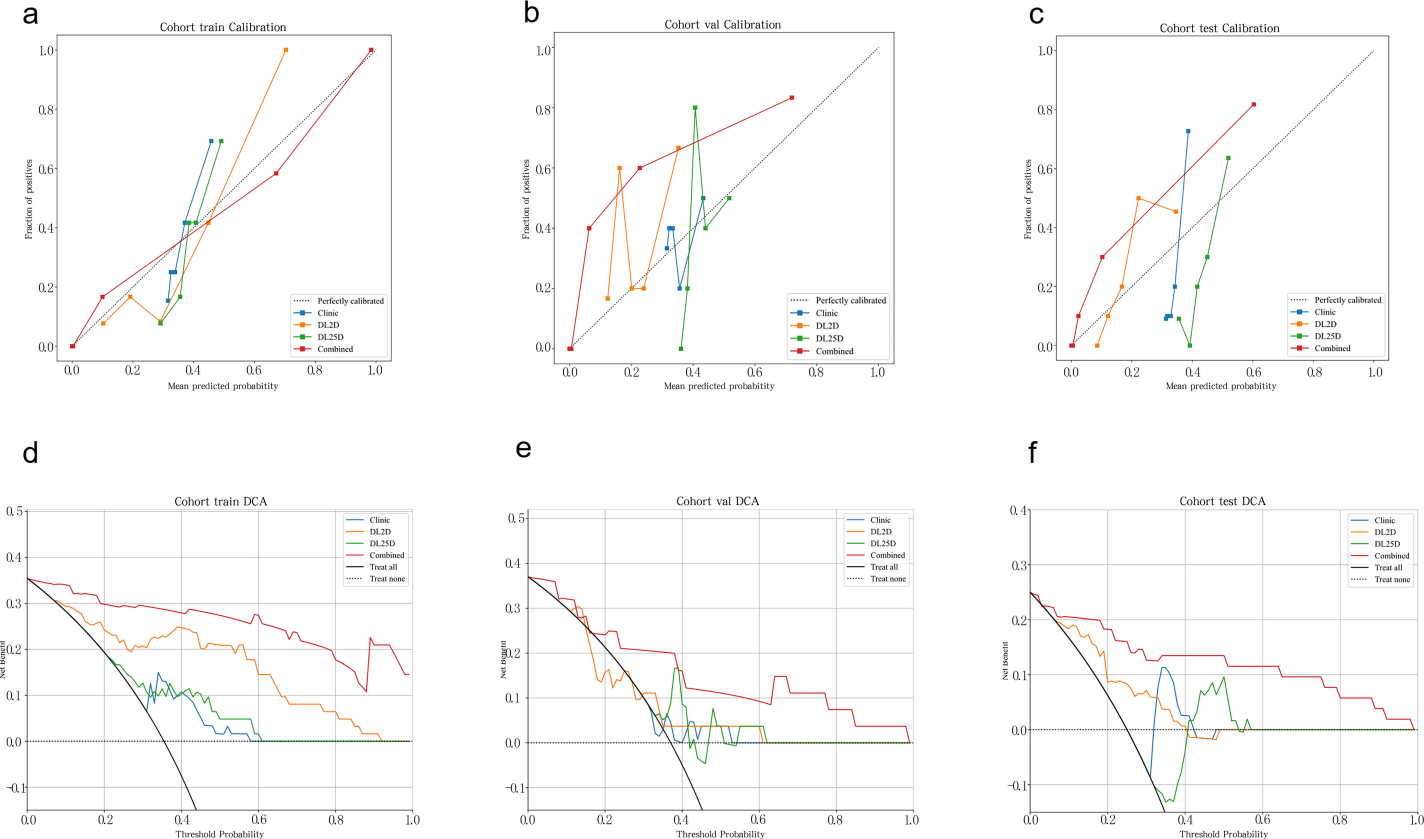
Calibration and DCA curves in the study cohorts. **(A–C)** illustrate the calibration curves for the models in the training, internal validation, and external validation cohorts, respectively; the black diagonal line represents ideal calibration, with greater proximity indicating superior model performance. **(D–F)** show the DCA curves for the models across cohorts. 2D, 2-dimensional; 2.5D, 2.5-dimensional; DCA, decision curve analysis; DL, deep learning.

To demonstrate the clinical superiority of the proposed MultiChannel_2.5D_DL model (intratumoral + 2-mm peritumoral ROI), we developed a combined model by integrating its output with the 2D_DL and clinical models via logistic regression. This model is aimed at differentiating benign from malignant breast nodules. The AUC values in the training cohort, internal validation cohort, and external validation cohort were 0.975 (95% CI: 0.945, 1.000), 0.929 (95% CI: 0.837, 1.000), and 0.949 (95% CI: 0.888, 1.000), respectively. The ROC curves for the combined model and individual models are shown in **Figures 4A–C**. The diagnostic performance metrics of this model are detailed in **Table 3**. The DeLong test indicated that the combined model significantly outperformed individual model signatures (mean p < 0.05) (**Figures 4D–F**). Additionally, the calibration curves for the combined model demonstrated good calibration in the training, internal validation, and external validation sets (**Figures 5A–C**). **Figure 5D-F** presents the DCA for all study cohorts. The combined model demonstrated considerable advantages in terms of predicted probabilities and consistently yielded greater net clinical benefit compared with the other model signatures, thereby underscoring its clinical utility. In the external validation cohort, the combined model showed numerically superior performance to BI-RADS assessment by senior radiologists using the ≥4B cutoff (AUC 0.949 [95% CI: 0.888, 1.000] vs 0.897 [95% CI: 0.808, 0.986], p=0.15); **Table 4**).

**Table 3:** Performances of the combined models in the study cohorts.

Model name	Set	Accuracy	AUC	Sensitivity	Specificity	PPV	NPV	F1
Combined	Training	0.935	0.975 (0.945-1.000)	0.909	0.950	0.909	0.950	0.909
	Internal validation	0.852	0.929 (0.837-1.000)	1.000	0.529	0.714	1.000	0.833
	External validation	0.923	0.949 (0.888-1.000)	0.846	0.949	0.846	0.949	0.846

AUC, area under the receiver; BI-RADS, Breast Imaging-Reporting and Data System; NPV, negative predictive value; PPV, positive predictive value; F1, F1-score.

**Table 4:** Diagnostic performance of the combined model compared with BI-RADS assessment by senior radiologists in the external validation cohort.

Model name	Accuracy	AUC	Sensitivity	Specificity	PPV	NPV	F1
Combined	0.923	0.949 (0.888-1.000)	0.846	0.949	0.846	0.949	0.846
US_BI-RADS (senior radiologists, ≥4B cutoff)	0.885	0.897 (0.808-0.986)	0.923	0.872	0.706	0.971	0.800

AUC, area under the receiver; NPV, negative predictive value; PPV, positive predictive value; F1, F1-score.

### 3.4 Grad-CAM visualization

**Figure 6:**
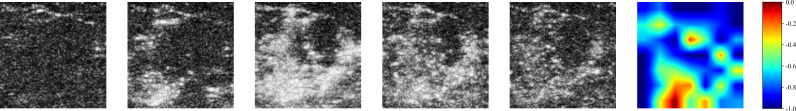
Grad-CAM visualizations of a representative sample, illustrating the MultiChannel 2.5D_DL model’s selective focus on image regions for prediction. Red areas denote heightened model attention, indicating enhanced prognostic value for response prediction. 2.5D, 2.5-dimensional; Grad-CAM, gradient-weighted class activation mapping; DL, deep learning.

To assess the recognition capability of the DL models, the Grad-CAM technique was applied for visual interpretation. As illustrated in **Figure 6**, Grad-CAM localized the most informative regions in the final convolutional layer for cancer type prediction. This approach enabled identification of image areas exerting the greatest influence on model decisions, thereby enhancing the interpretability of the DL framework.

## 4 Discussion

MultiChannel_2.5D_DL models typically process multiple adjacent 2D slices or channels as input, thereby capturing partial 3D context without the computational burden associated with full 3D models. These models often employ parallel 2D CNNs or transformer-based architectures to integrate information across slices or modalities (Hu et al., 2019; Alsaih et al., 2020; Kim et al., 2024; Niu et al., 2025). In contrast, 2D models process single slices or channels independently, which limits their ability to capture inter-slice correlations (Xiong et al., 2023; Niu et al., 2025; Tondji et al., 2025). In this study, we developed MultiChannel_2.5D_DL, 2D_DL, and clinical models based on B-mode US and CEUS images, along with clinical data from the primary tumor, to predict the malignancy of breast nodules. Our results demonstrated that the segmentation model incorporating a 2-mm peritumoral ROI yielded the optimal predictive performance; in this context, the MultiChannel_2.5D_DL model outperformed both the clinical model and the 2D DL model in the internal and external validation sets.

ROI delineation was performed independently by two experienced radiologists, with any discrepancies resolved by a senior radiologist with over 20 years of experience. Although quantitative inter-observer consistency metrics (such as the Dice coefficient or intraclass correlation coefficient) were not formally calculated in this study, the senior review process was implemented to minimize variability and enhance reproducibility.

Although no data augmentation or class-balancing techniques were employed, overfitting was mitigated through transfer learning with ImageNet-pretrained weights, early stopping was not required due to stable convergence, and external validation on an independent cohort provided robust evaluation despite the class imbalance.

In previous studies, MultiChannel_2.5D_DL models have outperformed 2D models in tasks such as tumor classification, organ segmentation, and disease subtype differentiation. For example, in glioma classification, the MultiChannel_2.5D_DL model achieved AUC values of 0.806–0.870, compared with lower performance for 2D models (Niu et al., 2025). In pancreas segmentation, the 2.5D model reached a Dice similarity coefficient of 75.1%, surpassing the 2D model (Tondji et al., 2025). These findings are consistent with the results of the present study.

Recent studies have demonstrated that MultiChannel_2.5D_DL models, particularly when integrated with clinical and radiomics features, can significantly enhance diagnostic accuracy and risk stratification across diverse applications. For example, in glioma subtyping, a MultiChannel_2.5D_DL model achieved AUC values of 0.806–0.870, while a stacking ensemble further improved performance to 0.855–0.904 (Niu et al., 2025). In hepatocellular carcinoma recurrence prediction, a combined 2.5D DL and clinical model attained AUCs up to 0.921 (Zhang et al., 2024). These findings are consistent with the results of the present study. In our study, we integrated the MultiChannel_2.5D_DL, 2D_DL, and clinical models to create a combined model, which was evaluated using an external validation cohort and exhibited the highest performance among all models assessed.

Previous studies have highlighted the broad application of DL in breast cancer diagnosis, with many models achieving high diagnostic performance. For example, Jiang et al. (2021) applied deep convolutional neural networks to ultrasound (US) images for molecular subtype classification, achieving accuracies ranging from 80.07% to 97.02% across four subtypes. Xiang et al. (2023) reported that a dual attention-based CNN reached expert-level performance in differentiating malignant from benign tumors, with AUCs up to 0.96 in external datasets. Similarly, Jia et al. (2023) demonstrated that DL models could noninvasively predict tumor-infiltrating lymphocyte levels from US images, with an AUC of 0.873 and an external validation accuracy of 79.50%, providing an alternative to traditional biopsy. Despite these advances, most existing approaches rely primarily on B-mode US, with limited exploration of CEUS. To our knowledge, the present study is the first to utilize a TIC dataset to identify key phases of contrast enhancement and to integrate MultiChannel_2.5D_DL for predictive modeling of breast nodules. In addition, we systematically compared the diagnostic performance of models based on tumor bounding boxes, intratumoral regions, and intratumoral regions expanded by 2-mm and 5-mm peritumoral margins.

CEUS employs microbubble contrast agents to improve the visualization of blood flow in both intratumoral and peritumoral regions, thereby yielding insights into tumor biology and aggressiveness (Zhao et al., 2017). Specific enhancement patterns, such as perfusion defects and peripheral hyperenhancement, represent critical features for differentiating benign from malignant lesions (Li et al., 2025). Malignant lesions commonly demonstrate an expansion of the enhancement area beyond the tumor boundaries as depicted on conventional ultrasound (Cai et al., 2013; Boca (Bene) et al., 2021). A recent study has shown that radiomics models incorporating features from both intratumoral and peritumoral regions outperform those based solely on intratumoral features, attaining high diagnostic accuracy (AUCs up to 0.949) and offering enhanced clinical decision support for the early detection of breast cancer (Li et al., 2025). Moreover, CEUS-based radiomics, particularly when integrating intratumoral and peritumoral features, facilitates the noninvasive prediction of breast cancer molecular subtypes, including luminal, HER2-positive, and triple-negative variants. These combined models achieve high accuracy (AUCs up to 0.956) in distinguishing luminal from non-luminal subtypes (Xu et al., 2025). In addition, ultrasound-based radiomics can discriminate among three distinct HER2 expression states (positive, low, and zero) with macro-AUCs up to 0.988, thereby aiding personalized decisions on HER2-targeted therapies (Luo et al., 2025).

The present study integrates DL methodologies with CEUS imaging to enhance breast cancer diagnosis. Notably, it innovatively employs MultiChannel_2.5D technology for training predictive models, yielding superior performance of the intratumoral plus peritumoral models over the intratumoral model alone in the training cohort, consistent with prior investigations. In the external validation cohort, the intratumoral model combined with a 2-mm ROI exhibited higher efficacy compared to combinations with a 5-mm ROI or the bounding box model. This disparity may arise from the relatively small diameters of the breast nodules included in this study, where extending the peritumoral distance beyond a certain threshold incorporates excessive irrelevant information; in contrast, the 2-mm ROI comprehensively captures peritumoral blood flow data without including substantial extraneous details. In our MultiChannel_2.5D_DL model for predicting malignancy in breast nodules, Grad-CAM visualizations revealed focal activations manifesting as irregular clusters and hotspots, with predominant emphasis on lesion boundaries. This saliency pattern highlights the model’s reliance on edge-related features, which are augmented by morphological intricacies and high-contrast gradients derived from dynamic perfusion variations in CEUS time-intensity curve images across five temporal phases. Specifically, these boundaries elicit robust gradient responses attributable to characteristic perfusion profiles, such as peripheral hyperenhancement or filling defects, thereby enhancing discriminative capability for malignancy identification. The 2.5D architecture effectively assimilates these multi-channel temporal inputs, extracting and fusing inter-phase contextual cues pertaining to blood flow dynamics. Consequently, model attention is directed toward boundary regions, where cross-phase temporal coherence yields resilient features that underpin improved diagnostic accuracy.

In summary, our results demonstrate that MultiChannel_2.5D_DL models, when combined with CEUS and an optimally defined peritumoral ROI, provide superior diagnostic performance compared with 2D and clinical models. Notably, the 2-mm peritumoral region proved most effective, whereas larger ROIs introduced redundant information that impaired model efficacy. These findings underscore the dual importance of advanced network architectures and precise ROI delineation, and suggest that systematic evaluation of peritumoral region size may further improve the reliability and generalizability of CEUS-based DL models in breast cancer diagnosis.

Although the difference did not reach statistical significance (p=0.15), the combined model demonstrated diagnostic performance comparable to that of senior radiologists using the ≥4B cutoff (AUC 0.949 vs. 0.897; **Table 4**). These findings highlight its potential as an AI-assisted decision support tool to augment radiologist performance in clinical practice.

This study has several limitations. First, the relatively small sample size (n=141 overall, with only 13 malignant nodules in the external validation cohort) may limit statistical power and generalizability. To mitigate this, we employed transfer learning with ImageNet-pretrained weights, external validation across two centers with different ultrasound systems, and rigorous cross-validation strategies. Nevertheless, future studies with larger, more balanced cohorts are warranted to further validate the model. Second, the two centers used different ultrasound systems and probe frequencies over a 6–8 year period. Although mechanical index and contrast agent dosage were standardized, residual differences in imaging parameters cannot be entirely excluded. Importantly, the multichannel 2.5D model primarily captured relative changes in time-intensity curves across ROIs rather than absolute grayscale intensity. Consequently, differences in probe frequency or system gain are expected to have minimal impact on model performance, as relative enhancement patterns remain consistent across platforms.

## 5 Conclusions

Our study demonstrates that the proposed MultiChannel_2.5D_DL model, which incorporates TIC-derived multichannel inputs and an optimally identified 2-mm peritumoral ROI, offers promising accuracy and generalizability when integrated with 2D_DL and clinical features into a combined model. This combined model demonstrated diagnostic performance comparable to that of senior radiologists, although the difference did not reach statistical significance. These findings highlight its potential as an AI-assisted decision support tool for CEUS-based breast nodule malignancy prediction, potentially assisting radiologists in reducing interobserver variability and unnecessary biopsies.

## Data Availability

The datasets presented in this article are not readily available because The de-identified dataset is not publicly available due to institutional data protection policies and ethical restrictions but can be accessed from the corresponding author upon reasonable request, subject to approval by the institutional ethics committee and execution of a formal data use agreement. Requests to access the datasets should be directed to kunsun, kunsun68@hotmail.com.
